# Bacterial Profile and Antimicrobial Susceptibility Patterns in Diabetic Foot Ulcers: A Cross‑Sectional Study in Bangladesh

**DOI:** 10.7759/cureus.99212

**Published:** 2025-12-14

**Authors:** Rinky Sharma, Abdul Ahad, Mohammad Asif Khan, Shaima Akter, Rabeya Yousuf, Md Muzammel Haque, Md Mushtahid Salam, Abdus Salam

**Affiliations:** 1 General Medicine, Bedfordshire Hospitals NHS Foundation Trust, Luton, GBR; 2 Microbiology and Veterinary Public Health, Chattogram Veterinary and Animal Sciences University, Chattogram, BGD; 3 Program Development, Base Care Foundation, London, GBR; 4 General and Specialist Medicine, Ashford and St. Peter's Hospitals NHS Foundation Trust, Surrey, GBR; 5 Diagnostic Laboratory Services, Hospital Canselor Tuanku Muhriz, Universiti Kebangsaan Malaysia, Kuala Lumpur, MYS; 6 Geriatric Medicine, Lincoln County Hospital, Lincoln, GBR; 7 Centre for Data Analytics and Society, Cathie Marsh Institute for Social Research, University of Manchester, Manchester, GBR; 8 Medical Education and Public Health, Faculty of Medicine, Widad University College, Kuantan, MYS

**Keywords:** antibiotic susceptibility testing, antimicrobial susceptibility testing, bacterial profile, bangladesh, cluster analysis, diabetes mellitus, diabetic foot ulcer

## Abstract

Introduction

Diabetic foot ulcers (DFUs) are a significant cause of amputation and mortality in low‑ and middle‑income countries. However, Bangladeshi data on the bacterial spectrum and antimicrobial susceptibility patterns remain scarce.

Methods

In a cross‑sectional study at Chattogram Diabetic General Hospital, Bangladesh, 106 adults with DFUs provided deep tissue specimens as per grading for aerobic culture. Bacterial isolates were identified by standard methods and tested against 16 antibiotics according to Clinical and Laboratory Standards Institute (CLSI) disc diffusion guidelines. Clinical and sociodemographic data were summarised descriptively; resistance patterns were visualised using heat‑map clustering.

Results

Culture was positive in 81.1% (86/106) of participants. Across all participants, *Staphylococcus aureus *(*S. aureus*) and *Escherichia coli *(*E. coli*) were co‑dominant (each 26.4%, 28/106), followed by *Klebsiella pneumoniae *(*K. pneumoniae*) and *Enterococcus *species (spp.) (each 11.3%, 12/106), and *Pseudomonas aeruginosa *(*P. aeruginosa*) (9.4%, 10/106); polymicrobial infection was 3.8% (4/106). Resistance was widespread. In the Gram‑positive panel, erythromycin showed the highest resistance (*S. aureus* 92.9%; *Enterococcus *spp.* *83.3%), with ampicillin/vancomycin/linezolid also high. In the Gram-negative panel, ampicillin, ciprofloxacin and cefuroxime carried heavy resistance burdens (reaching 100% in *P. aeruginosa*). By contrast, resistance to piperacillin-tazobactam and imipenem was low in *E. coli *(10.7% and 14.3%, respectively); in *K. pneumoniae* it was higher for piperacillin-tazobactam than imipenem (41.7% vs 16.7%); and in *P. aeruginosa *the pattern was reversed, with 10.0% resistant to piperacillin-tazobactam and 40.0% resistant to imipenem. Tigecycline retained 0% resistance across all taxa. Row‑wise clustering separated high‑ from lower‑resistance drug groups, making organism‑specific patterns immediately interpretable.

Conclusion

Gram‑negative organisms were more frequent overall, and resistance to several commonly used β‑lactams and fluoroquinolones was high. Tigecycline showed the best preserved activity. Empirical treatment should be guided by local data, prioritise agents with retained activity against both Gram‑positive and Gram‑negative pathogens, and be promptly narrowed once susceptibilities are available. Continued local surveillance and strong antimicrobial stewardship are essential to limit further resistance.

## Introduction

Diabetes mellitus (DM) is a chronic metabolic disorder that significantly disrupts insulin secretion and action, leading to various complications. Uncontrolled DM can damage the heart, kidneys, eyes, and nerves, resulting in serious outcomes such as myocardial infarction, stroke, blindness, nephropathy, neuropathy, diabetic foot ulcers (DFUs), and lower limb amputations [1]. DFUs are among the most common complications of diabetes, affecting approximately 6.3% of individuals globally, with a higher prevalence in males [2]. Alarmingly, one in three patients with diabetes develops a DFU in their lifetime, and more than half of these ulcers become infected [3]. These infections can lead to gangrene, osteomyelitis, and other life-threatening conditions, substantially increasing the risks of amputation and mortality [4].

Severe DFUs are associated with multiple underlying conditions, such as hyperglycaemia, peripheral neuropathy, impaired immune function, and peripheral arterial disease. Effective management requires early diagnosis and comprehensive wound care, including surgical debridement, correction of metabolic imbalances such as hyperglycaemia and arterial insufficiency, and the initiation of targeted antimicrobial therapy [5,6]. Identifying the causative microorganisms and initiating pathogen-specific antibiotic treatment are essential for appropriate clinical management.

Studies have shown that bacterial growth patterns and antimicrobial susceptibility vary significantly across regions [6,7], with antibiotic resistance closely linked to widespread antibiotic use [7]. Therefore, understanding local pathogen profiles and susceptibility patterns is vital for effective treatment [5] and essential for guiding therapeutic decisions, controlling infections, and reducing complications.

In Chattogram, Bangladesh, there is a notable gap in the literature regarding the microbiological profile and antimicrobial susceptibility of DFU infections. This study is one of the first studies from Chattogram, Bangladesh, a resource-constrained setting, to address the gap by characterising bacterial pathogens isolated from DFU patients and evaluating their antibiotic susceptibility profiles.

This article was previously presented as a meeting abstract at the Bangladeshi Doctors' UK National Conference on October 19, 2024.

## Materials and methods

We conducted a single-centre, hospital-based cross-sectional observational study with prospective enrolment at Chattogram Diabetic General Hospital, Chattogram, Bangladesh, over six months. Participants presenting with DFUs were enrolled in the study using consecutive sampling. The inclusion criteria were adult diabetic patients with DFUs who were willing to participate and provided written informed consent. Exclusion criteria included patients under 18 years of age, those unable or unwilling to provide consent, and those with foot ulcers of non-diabetic origin. Ethical approval for the study was obtained from the Research Cell and Ethical Committee of Chattogram Veterinary and Animal Sciences University (CVASU) (approval CVASU/Dir(R&E)EC/2020/165(7)).

Data collection

All participants were informed about the purpose, procedures, and significance of the study, and written informed consent was obtained before data and sample collection. All participants had type 2 diabetes mellitus documented in their medical records. In routine clinical practice at Chattogram Diabetic General Hospital, diabetes is diagnosed according to fasting plasma glucose ≥7.0 mmol/L and/or two-hour plasma glucose ≥11.1 mmol/L after a 75 g oral glucose load and/or HbA1c ≥6.5% [8]. Data were captured with a structured questionnaire (Appendix 1). The questionnaire was pilot-tested on 10 DFU patients for clarity; no wording changes were required. The questionnaire recorded (i) sociodemographic characteristics: age, sex, education, monthly household income in Bangladeshi taka (BDT), residence, and occupation; (ii) smoking history and body mass index (BMI); (iii) diabetes profile: duration of diabetes mellitus (DM) (self-reported), fasting blood sugar (FBS), DM treatment type, and medication adherence (assessed with a single question asked in Bengali: “Do you take your diabetes medicine exactly as prescribed by your doctor?”; respondents answering “Yes, every day as prescribed” were classified as regularly adherent, whereas “Sometimes missed doses” or “No (stopped or rarely takes it)” denoted irregular adherence); (iv) existing comorbidities, diabetes-related complications, and recent antibiotic exposure within the past month due to DFU (prescribed by a registered physician or taken without prescription); and (v) ulcer characteristics-Wagner DFU classification, ulcer surface area, and ulcer duration. Diabetes related comorbidities (hypertension, dyslipidaemia, cardiovascular disease, cerebrovascular disease) and complications (neuropathy, nephropathy, retinopathy, peripheral vascular disease) were extracted from medical records, where they had been previously diagnosed. Ulcer surface area was estimated by multiplying the mean maximal length by the mean maximal perpendicular width. Each dimension (maximal length and maximal perpendicular width) was measured three times in millimetres (mm) by the investigator, using a new sterile, disposable paper ruler for each reading. The mean of the three length measurements and the mean of the three width measurements were then multiplied to calculate the ulcer surface area, thereby reducing measurement error.

Specimen collection and processing

Deep tissue samples from DFUs were obtained based on their grading using sterile cotton swabs with firm, circular movements. The swabs were immediately placed into sterile Eppendorf tubes containing Stuart’s transport medium. The tubes were transported to the laboratory in an immersed state to maintain aseptic conditions. Samples were inoculated onto blood agar and MacConkey agar plates and incubated aerobically at 35 °C for 24-48 hours. Bacterial isolates were identified using standard microbiological techniques, and antibiotic susceptibility was tested using the disc diffusion method, following Clinical and Laboratory Standards Institute (CLSI) guidelines [9]. Standard reference strains, such as *Escherichia coli* (*E. coli*) American Type Culture Collection (ATCC) 25922 and *Staphylococcus aureus* (*S. aureus*) ATCC 25923, were included as quality control organisms in every batch of susceptibility testing. Due to resource constraints, anaerobic cultures and duplicate sampling to confirm polymicrobial isolates were not performed.

The antimicrobial susceptibility panel comprised the β‑lactam agents amoxicillin, ampicillin, oxacillin and piperacillin-tazobactam (penicillins); cefuroxime, ceftriaxone and cefepime (second‑, third‑ and fourth‑generation cephalosporins); imipenem (carbapenem); the fluoroquinolones ciprofloxacin and levofloxacin; the macrolide erythromycin; the glycopeptide vancomycin; the oxazolidinone linezolid; the polymyxin colistin; the aminoglycosides amikacin and gentamicin; and the glycylcycline tigecycline.

Statistical analysis

Statistical analyses were conducted using R (version 4.5.1; R Foundation for Statistical Computing, Vienna, Austria) with the tidyverse and ComplexHeatmap packages. Continuous variables are presented as mean ± standard deviation (SD); categorical variables are presented as frequencies (n) with percentages (%). For the heatmaps, each cell shows the percentage of resistant isolates for every organism-antibiotic pair. Hierarchical clustering was applied to rows (antibiotics) using Euclidean distance and complete linkage, generating dendrograms that group antibiotics with similar resistance patterns.

## Results

Sociodemographic characteristics

The study cohort comprised 106 individuals (mean ± SD age 53.8 ± 9.9 years; 56.6% male). Most participants had no formal education (37.7%), with a smaller proportion educated to at least Secondary School Certificate (SSC) level (24.5%) or higher (Table 1).

**Table 1:** Sociodemographic characteristics of participants with DFU (n = 106) ^*^Data are presented as n (%) unless otherwise specified. Abbreviations: BDT, Bangladeshi taka (1 BDT ≈ US$0.01); DFU, diabetic foot ulcer; HSC, Higher Secondary Certificate; SSC, Secondary School Certificate.

Sociodemographic characteristics	n (%)^*^
Age (years), mean ± SD	53.8 ± 9.9
Age (years)	
35–39	12 (11.3)
40–49	20 (18.9)
50–59	34 (32.1)
60–69	32 (30.2)
70–75	8 (7.5)
Sex	
Male	60 (56.6)
Female	46 (43.4)
Education	
No formal education	40 (37.7)
Primary	18 (17)
SSC	26 (24.5)
HSC	10 (9.4)
Graduate	8 (7.5)
Postgraduate	4 (3.8)
Monthly household income (BDT)	
< 10000	44 (41.5)
10000–20000	60 (56.6)
> 20000	2 (1.9)
Residence	
Rural	64 (60.4)
Urban	42 (39.6)
Occupation	
Unemployed	24 (22.6)
Mental Work	48 (45.3)
Physical Work	34 (32.1)

Smoking history and body mass index

Roughly one-third reported a history of smoking (35.8%). Half of the cohort (50.9%) fell within the normal body mass index (BMI) range (18.5-24.9 kg/m²); 35.8% were overweight (25-29.9 kg/m²) and 9.4% were underweight (< 18.5 kg/m²), while 3.8% were obese (≥ 30 kg/m²) (Table 2).

**Table 2:** Smoking history and BMI of participants with DFU (n = 106) Abbreviations: BMI, body mass index; DFU, diabetic foot ulcer.

Smoking history and BMI	n (%)
Ever Smoked	
No	68 (64.2)
Yes	38 (35.8)
Smoking status	
Never	68 (64.2)
≤ 10 years	14 (13.2)
11–20 years	6 (5.7)
21–30 years	10 (9.4)
31–40 years	4 (3.8)
41–50 years	4 (3.8)
BMI (kg/m^2^)	
< 18.5	10 (9.4)
18.5–24.9	54 (50.9)
25–29.9	38 (35.8)
≥ 30	4 (3.8)

Diabetes-related characteristics

All participants had type 2 DM. Nearly half the participants had lived with DM for 11-20 years (47.2%), while only 5.7% had a duration exceeding 20 years (Table 3). Mean ± SD FBS was 10.4 ± 4.3 mmol/L, and one-third of patients (34%) had markedly elevated levels (≥ 11.1 mmol/L) of FBS. Oral hypoglycaemic agents were the most common treatment (35.8%), followed by insulin alone (20.8%), while 35.8% used both modalities. Two-thirds reported regular adherence to DM treatment.

**Table 3:** Diabetes-related characteristics of participants with DFU (n = 106) ^*^Data are presented as n (%) unless otherwise specified. Abbreviations: DM, diabetes mellitus; DFU, diabetic foot ulcer; FBS, fasting blood sugar; OHA, oral hypoglycaemic agents.

Diabetes profile	n (%)^*^
DM duration	
≤ 5 years	20 (18.9)
6–10 years	30 (28.3)
11–20 years	50 (47.2)
21–30 years	6 (5.7)
FBS (mmol/L), mean ± SD	10.4 ± 4.3
FBS (mmol/L)	
≤ 7.2	34 (32.1)
> 7.2 and < 11.1	36 (34)
≥ 11.1	36 (34)
DM treatment type	
No DM medicine	8 (7.5)
OHA	38 (35.8)
Insulin	22 (20.8)
OHA and Insulin	38 (35.8)
DM treatment adherence history	
Not applicable	8 (7.5)
Irregular	28 (26.4)
Regular	70 (66)

Existing comorbidities and complications

Most participants had at least one comorbidity (77.4%). Hypertension was the most prevalent (62.3%), followed by dyslipidaemia (47.2%) and cardiovascular disease (30.2%), while cerebrovascular disease was present in 11.3% of the participants (Table 4).

**Table 4:** Existing comorbidities among participants with DFU (n = 106) Abbreviation: DFU, diabetic foot ulcer.

Existing comorbidities	n (%)
Comorbidity status	
None	24 (22.6)
≥ 1	82 (77.4)
Hypertension	
No	40 (37.7)
Yes	66 (62.3)
Dyslipidaemia	
No	56 (52.8)
Yes	50 (47.2)
Cardiovascular disease	
No	74 (69.8)
Yes	32 (30.2)
Cerebrovascular disease	
No	94 (88.7)
Yes	12 (11.3)

Most participants had at least one diabetes-related complication (77.4%). Neuropathy was the most common (62.3%), followed by nephropathy (37.7%) and retinopathy (15.1%). Peripheral vascular disease and prior amputation were documented in 15.1% and 11.3% of participants, respectively. Seventeen percent of the participants had taken an antibiotic within the past month without a prescription from a registered physician (Table 5).

**Table 5:** Existing diabetes-related complications and recent antibiotic exposure among participants with DFU (n = 106) Abbreviation: DFU, diabetic foot ulcer.

Existing complications and recent antibiotic exposure	n (%)
Complication status	
None	24 (22.6)
≥ 1	82 (77.4)
Neuropathy	
No	40 (37.7)
Yes	66 (62.3)
Nephropathy	
No	66 (62.3)
Yes	40 (37.7)
Retinopathy	
No	90 (84.9)
Yes	16 (15.1)
Peripheral vascular disease	
No	90 (84.9)
Yes	16 (15.1)
Amputation	
No	94 (88.7)
Yes	12 (11.3)
Antibiotic exposure within the past month due to DFU	
No	22 (20.8)
Yes – Prescribed by a registered physician	66 (62.3)
Yes – Not prescribed by a registered physician	18 (17.0)

Ulcer characteristics and bacteriology

Half of the ulcers were Wagner grade 1 (50.9%); grades 0 and 2 accounted for 17% and 15.1%, respectively, while severe lesions (grades 3-5) comprised 17%. Mean ± SD ulcer surface area was 32.2 ± 37.6 mm², and 75.5% of ulcers measured ≤ 50 mm². Ulcers were present for a mean ± SD of 18.4 ± 14.9 days; 54.7% had existed for ≤ 14 days, 35.8% for 15-30 days, and 9.4% for > 30 days (Table 6).

**Table 6:** DFU characteristics of participants (n = 106) ^*^Data are presented as n (%) unless otherwise specified. ^†^Wagner DFU classification: Grade 0, no ulcer in a high‑risk foot; Grade 1, superficial ulcer involving full skin thickness but not underlying tissues; Grade 2, deep ulcer penetrating to ligaments and muscles without bone involvement or abscess; Grade 3, deep ulcer with cellulitis or abscess, often with osteomyelitis; Grade 4, localized gangrene; Grade 5, extensive gangrene of the whole foot. Abbreviation: DFU, diabetic foot ulcer.

DFU characteristics	n (%)^*^
Wagner ulcer classification^†^	
Grade 0	18 (17)
Grade 1	54 (50.9)
Grade 2	16 (15.1)
Grade 3	4 (3.8)
Grade 4	12 (11.3)
Grade 5	2 (1.9)
Ulcer surface area (mm^2^), mean ± SD	32.2 ± 37.6
Ulcer surface area (mm^2^)	
≤ 50	80 (75.5)
51–100	18 (17)
101–170	8 (7.5)
Ulcer duration (days), mean ± SD	18.4 ± 14.9
Ulcer duration (days)	
≤ 14	58 (54.7)
15–30	38 (35.8)
> 30 and ≤ 60	10 (9.4)

**Figure 1:**
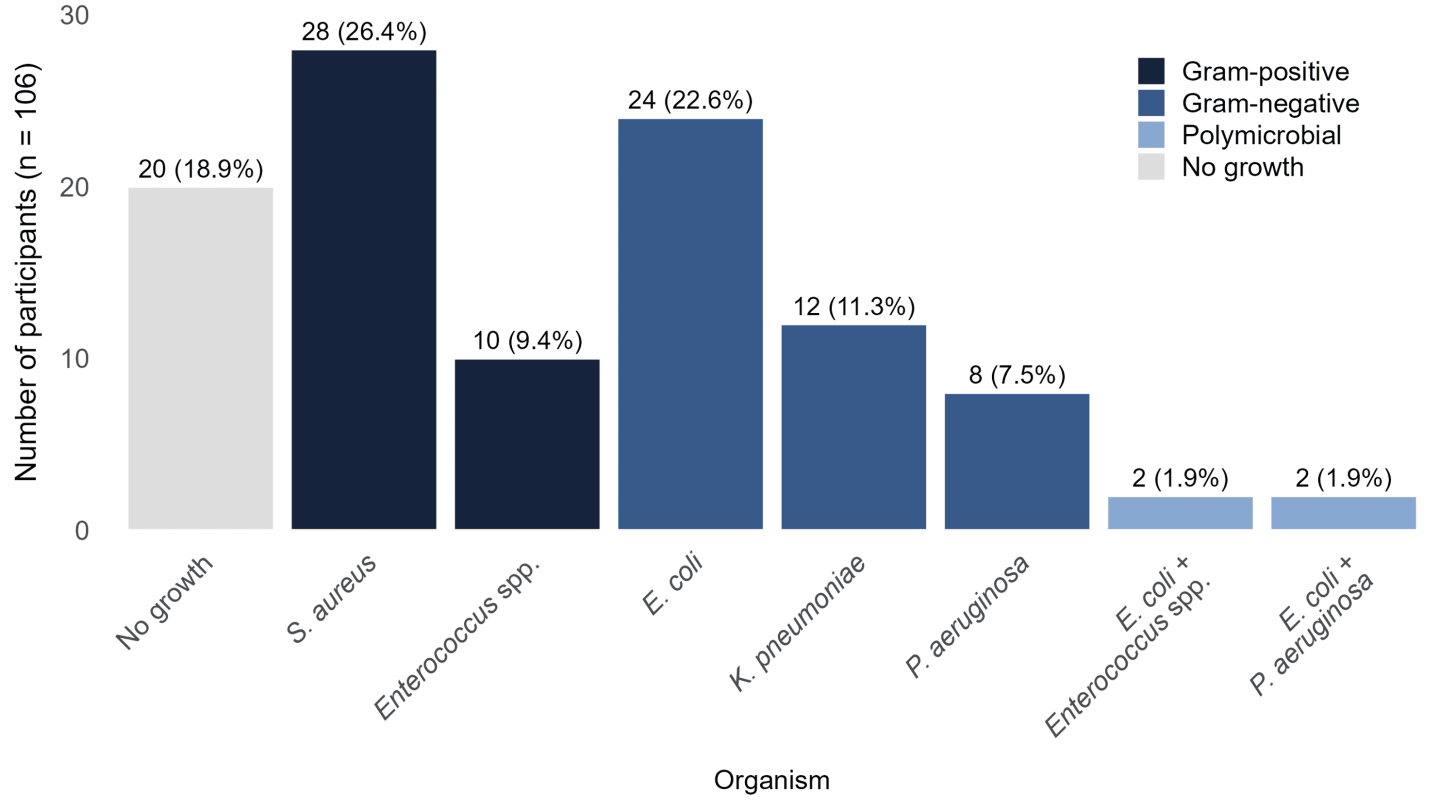
Frequency of DFU culture results by organism Abbreviation: DFU, diabetic foot ulcer.

​​​​Cultures yielded bacteria in 86 (81.1%) of 106 cases, while 20 (18.9%) showed no growth. Of the positive cultures, 82 were monomicrobial and four were polymicrobial. The most frequent organisms were *S. aureus* and *E. coli*, each isolated from a total of 28 (26.4%) participants. These were followed by *Klebsiella pneumoniae (K. pneumoniae) *and *Enterococcus *species (spp.), each found in 12 (11.3%) participants, and *Pseudomonas aeruginosa *(*P. aeruginosa*), found in 10 (9.4%) participants. While all *S. aureus *and *K. pneumoniae *isolates were from monomicrobial infections, the other organisms were also present in polymicrobial cultures. The four polymicrobial infections consisted of *E. coli *with *Enterococcus* spp. (n=2) and *E. coli *with *P. aeruginosa* (n=2). Overall, Gram-negative organisms predominated (Figure 1).

Antimicrobial susceptibility test

Gram-Positive Isolates

Table 7 shows that tigecycline retained 100% activity against both *S. aureus* and *Enterococcus* spp. *S. aureus* also remained highly susceptible to levofloxacin (78.6%), whereas erythromycin (7.1%) was largely ineffective. *Enterococcus *spp. exhibited high susceptibility to piperacillin-tazobactam (83.3%), levofloxacin (83.3%), but only 16.7% were susceptible to vancomycin.

**Table 7:** Antimicrobial susceptibility pattern of Gram-positive and Gram-negative isolates from DFUs Data are presented as n (%) per organism-antibiotic pair. Abbreviations: DFU, diabetic foot ulcer; S, Susceptible.

	Gram-positive		Gram-negative			
	*S. aureus* (n = 28)	*Enterococcus *spp. (n = 12)	*E. coli* (n = 28)	*K. pneumoniae* (n =12)	*P. aeruginosa *(n = 10)	
Antibiotic	S, n (%)	S, n (%)	S, n (%)	S, n (%)	S, n (%)	
Amikacin	-	-	10 (35.7%)	6 (50.0%)	2 (20.0%)	
						Amoxicillin	16 (57.1%)	8 (66.67%)	12 (42.9%)	6 (50.0%)	0 (0.0%)	
Ampicillin	11 (39.3%)	2 (16.67%)	4 (14.3%)	2 (16.7%)	0 (0.0%)	
Cefepime	-	-	18 (64.3%)	10 (83.3%)	4 (40.0%)	
						Ceftriaxone	-	-	6 (21.4%)	6 (50.0%)	4 (40.0%)	
Cefuroxime	-	-	8 (28.6%)	4 (33.3%)	0 (0.0%)							
						Ciprofloxacin	14 (50.0%)	4 (33.3%)	12 (42.9%)	2 (16.7%)	0 (0.0%)	
Colistin	-	-	18 (64.3%)	12 (100.0%)	6 (60.0%)	
						Erythromycin	2 (7.1%)	2 (16.7%)	8 (28.6%)	2 (16.7%)	4 (40.0%)	
Gentamicin	15 (53.6%)	8 (66.7%)	14 (50.0%)	2 (16.7%)	0 (0.0%)	
Imipenem	15 (53.6%)	8 (66.7%)	22 (78.6%)	10 (83.3%)	2 (20.0%)	
Levofloxacin	22 (78.6%)	10 (83.3%)	22 (78.6%)	4 (33.3%)	6 (60.0%)	
Linezolid	8 (28.6%)	5 (41.7%)	-	-	-	
						Piperacillin-Tazobactam	20 (71.4%)	10 (83.3%)	20 (71.4%)	6 (50.0%)	8 (80.0%)	
Tigecycline	28 (100.0%)	12 (100.0%)	26 (92.9%)	8 (66.7%)	6 (60.0%)	
Vancomycin	12 (42.9%)	2 (16.7%)	-	-	-	

Gram-Negative Isolates

Among the Gram-negative isolates (Table 7), *E. coli* was most susceptible to tigecycline (92.9%), levofloxacin (78.6%), imipenem (78.6%) and piperacillin-tazobactam (71.4%); *K. pneumoniae* retained full susceptibility to colistin (100.0%) and high susceptibility to cefepime and imipenem (83.3% each), while tigecycline covered 66.7% of isolates; ciprofloxacin was susceptible in only 16.7% of isolates. *P. aeruginosa* exhibited its greatest susceptibility to piperacillin-tazobactam (80.0%) and moderate activity to tigecycline, levofloxacin, and colistin (60.0% each); most other agents achieved ≤ 50.0% susceptibility.

Heatmap and hierarchical clustering

Gram-Positive Isolates

**Figure 2:**
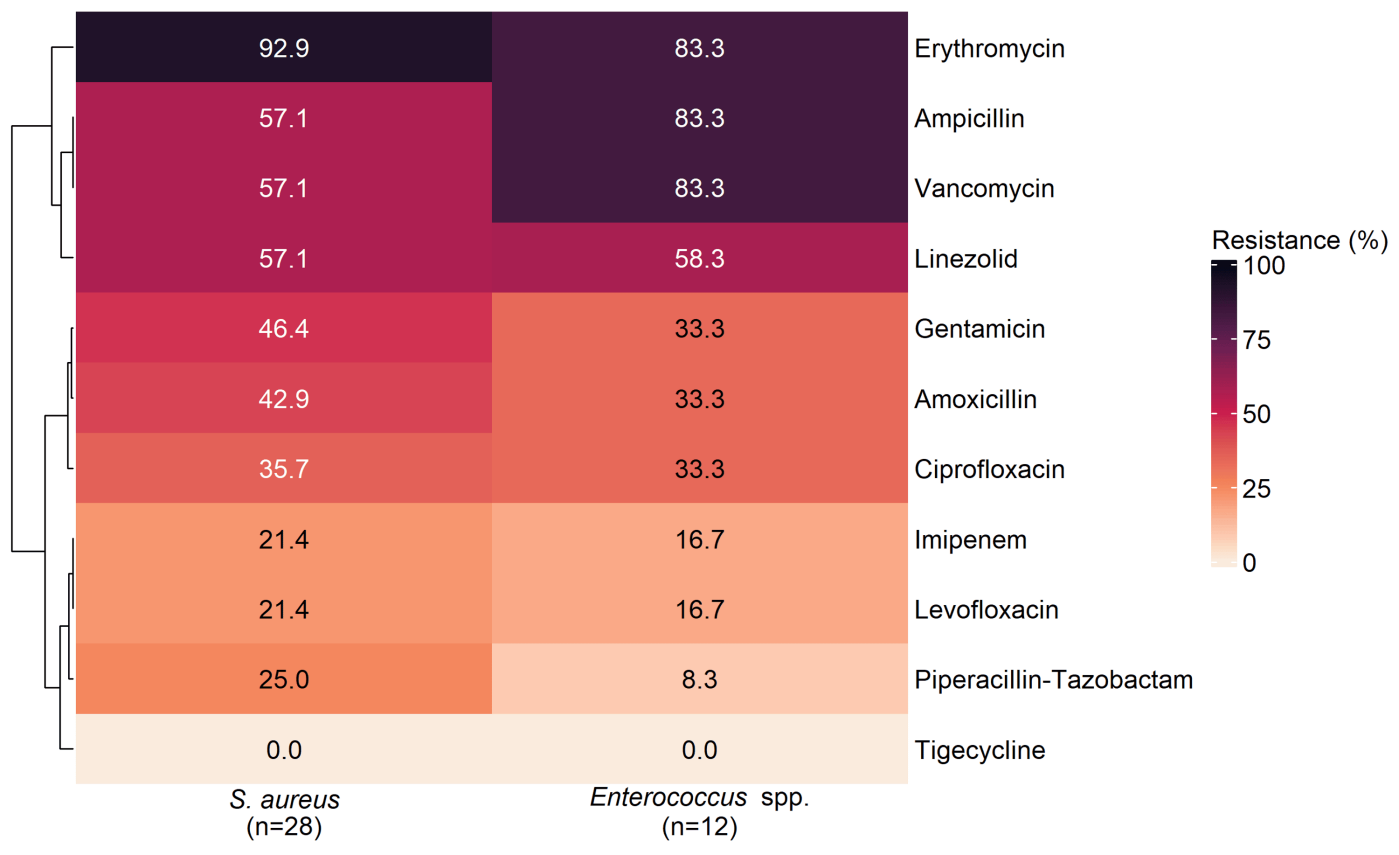
Heatmap and hierarchical clustering (dendrogram) of antibiotic resistance among Gram-positive DFU isolates Data are presented as % of Gram-positive DFU isolates for each organism-antibiotic pair. Abbreviation: DFU, diabetic foot ulcer.

Figure 2 depicts a clustered heatmap of percentage resistant isolates for 11 antibiotics tested against *S. aureus *(n = 28) and *Enterococcus *spp. (n = 12). Rows (antibiotics) are hierarchically clustered to group similar resistance profiles. The percentage of resistant isolates for each antibiotic is represented by color intensity, where darker shades indicate higher resistance, and the exact resistance percentage values are presented within each cell. The row dendrogram separated the antibiotics into three groups. A high‑resistance block comprised erythromycin (92.9% in *S. aureus*; 83.3% in *Enterococcus *spp*.*), alongside ampicillin, vancomycin and linezolid (each 57.1% in *S. aureus*; 83.3%, 83.3% and 58.3% in *Enterococcus *spp*.*). An intermediate block included gentamicin, amoxicillin and ciprofloxacin (46.4%, 42.9% and 35.7% in *S. aureus*; each 33.3% in *Enterococcus *spp*.*). A lower‑resistance block contained imipenem, levofloxacin and piperacillin-tazobactam (21.4%, 21.4% and 25.0% in *S. aureus*; 16.7%, 16.7% and 8.3% in *Enterococcus *spp*.*), while tigecycline showed 0.0% resistance in both organisms.

Gram-Negative Isolates

**Figure 3:**
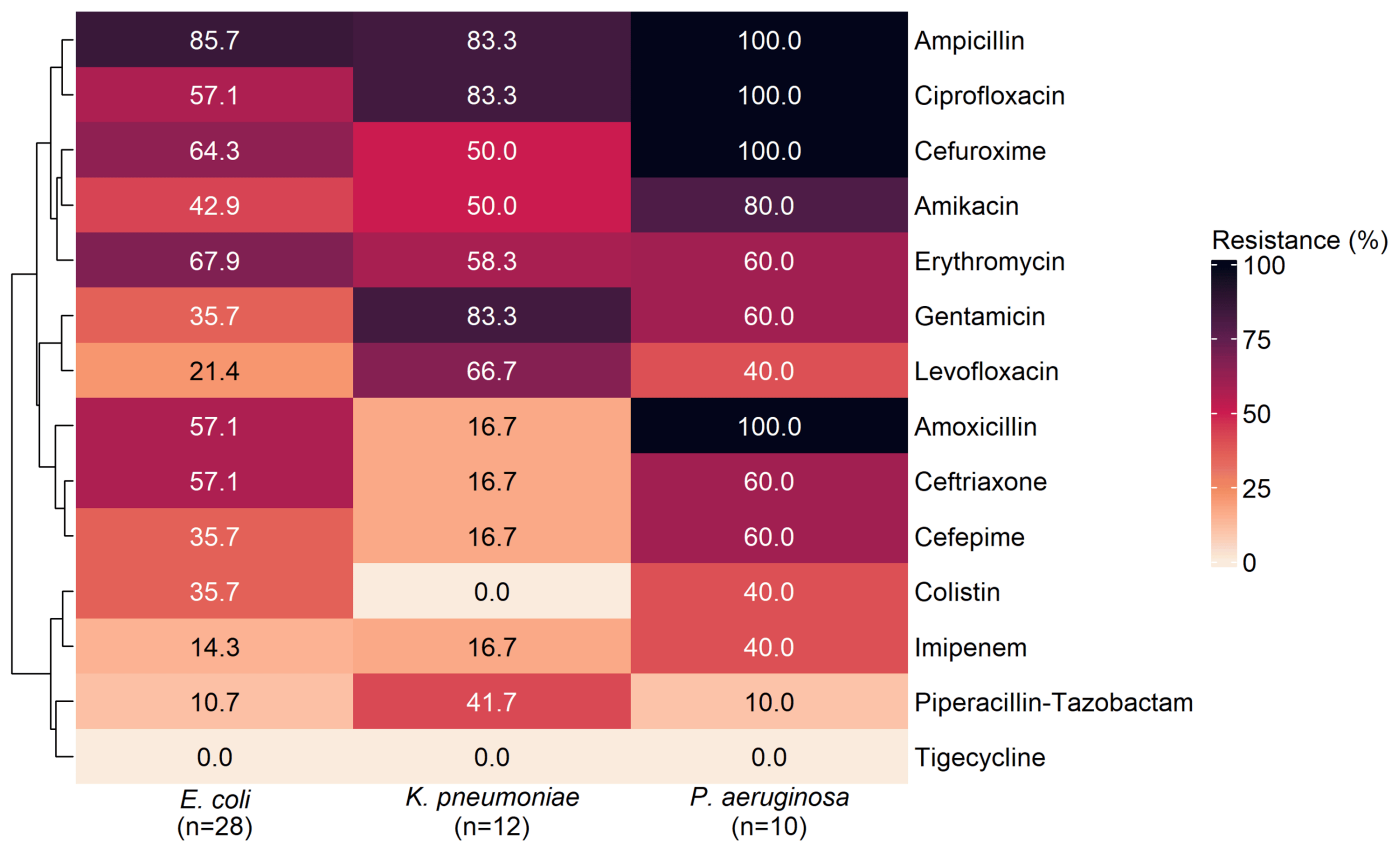
Heatmap and hierarchical clustering (dendrogram) of antibiotic resistance among Gram-negative DFU isolates Data are presented as % of Gram-negative DFU isolates for each organism-antibiotic pair. Abbreviation: DFU, diabetic foot ulcer.

Figure 3 presents a clustered heatmap of percentage resistant isolates for 14 antibiotics tested against *E. coli *(n = 28), *K. pneumoniae* (n = 12) and *P. aeruginosa *(n = 10)*. *The dendrogram arranged the drugs into blocks spanning higher to lower resistance. A high‑resistance block included ampicillin, ciprofloxacin and cefuroxime (*E. coli*: 85.7%, 57.1%, 64.3%; *K. pneumoniae*: 83.3%, 83.3%, 50.0%; *P. aeruginosa*: 100.0% for all three), with amikacin and erythromycin adjacent (*E. coli*: 42.9%, 67.9%; *K. pneumoniae*: 50.0%, 58.3%; *P. aeruginosa*: 80.0%, 60.0%). A mixed/intermediate block comprised gentamicin and levofloxacin (*E. coli*: 35.7%, 21.4%; *K. pneumoniae*: 83.3%, 66.7%; *P. aeruginosa*: 60.0%, 40.0%), together with amoxicillin, ceftriaxone and cefepime (*E. coli*: 57.1%, 57.1%, 35.7%; *K. pneumoniae*: each 16.7%; *P. aeruginosa*: 100.0%, 60.0%, 60.0%). The lower‑resistance block contained colistin (*E. coli*:* *35.7%; *K. pneumoniae*: 0.0%; *P. aeruginosa*: 40.0%), imipenem (*E. coli*: 14.3%, *K. pneumoniae*: 16.7%, *P. aeruginosa*: 40.0%) and piperacillin-tazobactam (*E. coli*: 10.7%, *K. pneumoniae*: 41.7%, *P. aeruginosa*: 10.0%), with tigecycline at 0.0% across all three organisms.

Across Figures 2, 3, tigecycline showed 0% resistance in every isolate, whereas ampicillin and several broad‑spectrum β‑lactams/fluoroquinolones carried high resistance burdens-particularly among Gram‑negative organisms and most prominently in *P. aeruginosa*, which reached 100% for multiple antibiotics.

## Discussion

Principal findings

This single‑centre study is the first to characterise DFU microbiology and antimicrobial susceptibility in Chattogram, Bangladesh. Four observations stand out. First, *S. aureus* and *E. coli* were co-dominant pathogens, followed by *K. pneumoniae* and *Enterococcus* spp., and *P. aeruginosa*, all common organisms found in DFUs [10]. Second, resistance was widespread. Among Gram-positive isolates, erythromycin showed the highest resistance, followed by ampicillin, vancomycin, and linezolid. Among Gram-negative organisms, ampicillin, ciprofloxacin, and cefuroxime had the greatest resistance, particularly in *P. aeruginosa*, which was 100% resistant to all three antibiotics. Finally, the clustered heatmaps separated high‑resistance blocks from lower‑resistance blocks, and tigecycline showed 0% resistance across all taxa.

Participant characteristics

This study revealed that male patients are more likely to develop DFUs than female patients, aligning with previous research findings [4,11,12]. However, another study [6] also found a predominance of DFU in females, indicating that this complication can affect individuals regardless of gender.

The highest incidence of DFU was found in those 50-59 years (32.1%), followed by 60-69 years (30.2%) and 40-49 years (18.9%). Other research found a similar trend with the highest occurrence in the 50-59 years age group (35.82%), followed by 60-69 years (27.34%), and 40-49 years (17.29%) [13].

Wagner DFU classification system found that the majority of the DFUs were classified as grade 1 (50.9%), followed by grade 2 (15.1%) and grade 4 (11.3%), indicating that most ulcers are of the less severe type. A study conducted in Bangladesh also found that most ulcers were classified as grade 2, followed by grades 3 and 4 [6]. In this present study, 86 (81.1%) of wound cultures found growth of an organism.

Our cohort carried a substantial burden of macro- and micro-vascular disease. 62.3% of participants were hypertensive and 47.2% had dyslipidaemia (Table 4), proportions comparable to the DFU study in other countries [14,15]. These comorbidities impair lower-limb perfusion, slow granulation, and restrict antibiotic options that require adequate renal clearance. Chronic complications of diabetes were also common (Table 5): 62.3% of patients had neuropathy, 37.7% had nephropathy, and 15.1% had retinopathy. Neuropathy helps explain why more than half the ulcers were Wagner grade 1: painless micro-trauma goes unnoticed until superficial infection develops. Conversely, nephropathy mandates caution with aminoglycosides and prompts preferential use of agents that are not primarily renally excreted. Taken together, this comorbidity profile emphasises the importance of integrated risk management alongside antimicrobial stewardship in DFU care.

Comparison with previous literature

The co-dominance of *S. aureus* and *E. coli* in our study reflects a complex picture consistent with regional findings. The high prevalence of *S. aureus *mirrors its species-level dominance reported in Bangladesh [12,16], Iran [17], China [11], Pakistan [18], Malaysia [19], and a recent global meta-analysis [20]. However, the equal prevalence of *E. coli* contributes to the second key finding: when isolates are pooled, our cohort was overwhelmingly Gram-negative. This Gram-negative predominance has been documented in nearby Malaysia [19,21,22], Pakistan [14,18], and India [23,24], and stands in contrast to more developed nations where Gram-positive organisms typically dominate DFU infections [20]. Geographic differences in healthcare infrastructure [20] and sanitation practices that facilitate faecal‑flora hand contamination [21] may partly explain these patterns.

While previous hospital-based studies have reported very high polymicrobial infection rates of up to 85% [14,22], such infections were uncommon in our study (3.8%). Nonetheless, Gram-negative bacteria like *E. coli* remained central to the mixed cultures that were detected.

In addition to the geographical variation in the predominance of the type of bacteria found in DFU, contradicting antimicrobial susceptibility results are also not uncommon [11,17]. While our study found *S. aureus* susceptibility to linezolid was 28.6%, a study in Pakistan reported a susceptibility of 82.8% [14]. Such divergence may reflect unregulated antibiotic access and repeated hospitalisation, both drivers of resistance in chronic wounds [17].

The sale of systemic antibiotics without a registered physician’s prescription is prohibited in Bangladesh under the National Drug Policy [25]. Nonetheless, 17% of the participants in our study gave a history of taking an antibiotic in the last month due to DFU without a prescription from a registered physician. A nationwide survey conducted in Bangladesh found that 50.9% of all antibiotics were dispensed without prescription in pharmacies [25]. Such figures are worrisome, as in low‑ and middle‑income countries, one of the main drivers of growing antimicrobial resistance is the sale of antibiotics without a prescription from a registered physician. Therefore, to combat the rising rate of antibiotic resistance and its serious complications, such as amputation from a superficial DFU, not only is appropriate antibiotic therapy crucial, but it is equally vital that policies and laws preventing the irrational sale of antibiotics without a physician’s prescription are strictly implemented and enforced.

Clinical implications

Our findings show that resistance was widespread among commonly used antibiotics. In the Gram‑positive panel, erythromycin showed the highest resistance (92.9% in *S. aureus*, 83.3% in *Enterococcus* spp.), and ampicillin/vancomycin/linezolid were also high. Notably, only 16.7% of *Enterococcus* spp. isolates were susceptible to vancomycin; this unusually low susceptibility should be interpreted with caution. In the Gram‑negative panel, ampicillin, ciprofloxacin and cefuroxime carried the heaviest burdens (reaching 100% in *P. aeruginosa*), whereas resistance to piperacillin-tazobactam and imipenem was low in *E. coli* (10.7% and 14.3%, respectively); in *K. pneumoniae* resistance was higher for piperacillin-tazobactam than imipenem (41.7% vs 16.7%); and in *P. aeruginosa* the pattern was reversed, with 10.0% resistant to piperacillin-tazobactam and 40.0% resistant to imipenem. Aminoglycosides showed mixed performance (e.g., gentamicin lower resistance in *E. coli* but high in *K. pneumoniae* and *P. aeruginosa*), demonstrating that empirical value is organism‑dependent, not class‑wide. Tigecycline retained 0% resistance across all taxa and marks the best‑preserved activity in our panel.

These patterns argue against routine empirical use of the high‑resistance group in this setting and support culture‑directed therapy with early de‑escalation. When *P. aeruginosa* is suspected, empirical regimens should include targeted anti‑pseudomonal coverage, given the extreme resistance to several first‑line agents here, with tailoring once susceptibilities return.

Where polymicrobial infection is likely, combination regimens guided by local susceptibility data should be considered. Research indicates that relying on a single antibiotic often falls short of adequately addressing the diverse array of bacteria that can be present in DFUs [26]. Compounding this issue, the ineffective penetration of antibiotics into lower limb tissue due to peripheral arterial disease significantly hampers treatment efficacy [27]. As a result, the strategic selection of antibiotics is important and vital for effectively managing DFUs and preventing severe complications.

Most common antibiotics prescribed in DFUs

Antibiotic selection in DFU should be guided by infection severity, suspected pathogens and local susceptibility data [28]. For mild infections, oral flucloxacillin is often recommended as first-line therapy, with clarithromycin, doxycycline or erythromycin (in pregnancy) as alternatives for patients with penicillin allergy or for whom flucloxacillin is not suitable to use [28]. For moderate or severe infections, recommended regimens include oral or intravenous flucloxacillin plus gentamicin and/or metronidazole, co-amoxiclav with or without gentamicin, or intravenous ceftriaxone combined with metronidazole [28]. When *P. aeruginosa* is suspected, piperacillin-tazobactam, or clindamycin with ciprofloxacin and/or gentamicin may be used, and if methicillin-resistant *S. aureus* (MRSA) is suspected or confirmed, vancomycin, teicoplanin, or linezolid is added [28].

Causes of resistance to commonly used antibiotics in DFUs 

Resistance to commonly used antibiotics in DFU is largely attributed to inappropriate antibiotic use, including unnecessary prescriptions, subtherapeutic dosing and prolonged or repeated courses [29]. Comorbidities such as chronic kidney disease, heart failure and microvascular disease may further promote resistance by altering antibiotic pharmacokinetics and increasing the need for repeated antibiotic exposure. Inadequate antibiotic selection and dosing can also favour biofilm formation and persistence in chronic ulcers, allowing bacteria to survive and acquire additional resistance mechanisms [29].

The inappropriate use of antibiotics contributes to both rising antimicrobial resistance and increased healthcare costs. Therefore, antibiotics are not recommended for clinically uninfected DFUs to prevent infection or promote wound healing. When classic signs of infection are masked by ischaemia and neuropathy, secondary indicators, such as serous exudate, delayed wound healing, friable or discoloured granulation tissue, malodour, or wound deterioration, may serve as evidence of infection [30].

Novel strategies to overcome antibiotic resistance 

The rise of antibiotic-resistant microorganisms has also prompted exploration of novel strategies to overcome resistance [31]. These include (i) discovery of new antibiotics by chemically modifying existing agents and screening large small-molecule libraries; (ii) enhancing the efficacy of existing antibiotics through more sophisticated drug-delivery systems or by modulating bacterial metabolism to increase susceptibility; and (iii) alternative or adjunctive therapies such as bacteriophages and their endolysins, anti-biofilm agents, probiotics, nanomaterials with intrinsic antimicrobial activity, and vaccine- or antibody-based approaches [31]. These modalities are being actively explored for DFU infections [32-34].

Strengths and limitations

This is one of the first studies to characterize DFU microbiology in Chattogram, the port city of Bangladesh. As such, the findings can make a notable contribution to local and national diabetic foot care guidelines. Limitations include the absence of anaerobic cultures and the lack of duplicate sampling to confirm polymicrobial growth due to resource constraints. Consequently, the prevalence of polymicrobial infection is likely underestimated.

A key strength is the use of hierarchical clustering with heatmaps. This moves beyond simple frequency tables to reveal complex resistance patterns that are immediately interpretable for clinicians and directly useful for empirical therapy decisions. The study also benefits from the use of deep tissue sampling, which improves the accuracy of microbiological findings. 

Taken together, this study provides valuable insights into the bacterial spectrum and antibiotic resistance patterns of diabetic foot infections in a specialised clinical setting. Although conducted at a single diabetic hospital and therefore potentially subject to selection bias - reflecting the referral patterns, case mix, and microbiological practices of this institution - it nevertheless offers an important reference point for similar centres in resource-limited contexts. To strengthen the generalisability of these findings and capture wider geographic and microbial diversity, larger multi-centre surveillance studies incorporating both aerobic and anaerobic cultures are needed.

## Conclusions

In Chattogram, Bangladesh, bacteria isolated from DFUs showed widespread resistance to commonly used β‑lactams and fluoroquinolones. Across taxa, tigecycline retained complete activity (0% resistance). Cefepime and colistin provided intermediate coverage in *E. coli *and *P. aeruginosa*, and levofloxacin was variably active. *P. aeruginosa *displayed the highest overall resistance, whereas *S. aureus *and *E. coli *were co‑dominant among isolates; overall, Gram‑negative organisms were more frequent than Gram‑positive. Row‑wise hierarchical clustering separated a high‑resistance group (e.g., ampicillin, ciprofloxacin, cefuroxime in Gram‑negatives; erythromycin and several agents in Gram‑positives) from a lower‑resistance group (notably piperacillin-tazobactam and imipenem for *E. coli*/*K. pneumoniae*), supporting culture‑directed, rapidly de‑escalated therapy and targeted anti‑pseudomonal coverage when *P. aeruginosa *is suspected. These findings underline the need for ongoing local surveillance and stewardship interventions to limit unnecessary broad‑spectrum use and slow further resistance escalation.

## Figures and Tables

**Figure 4:**
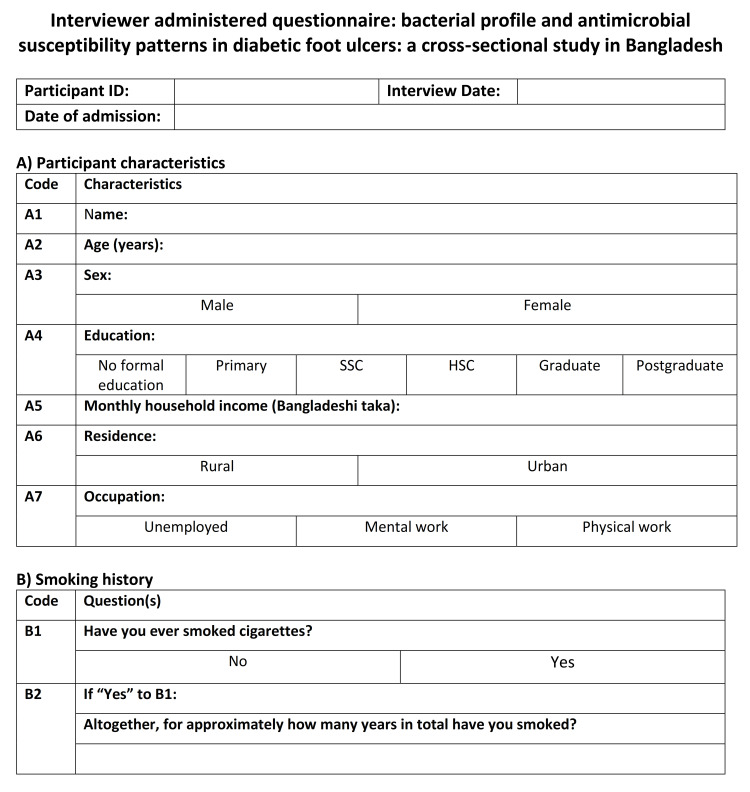
Study questionnaire page one

**Figure 5:**
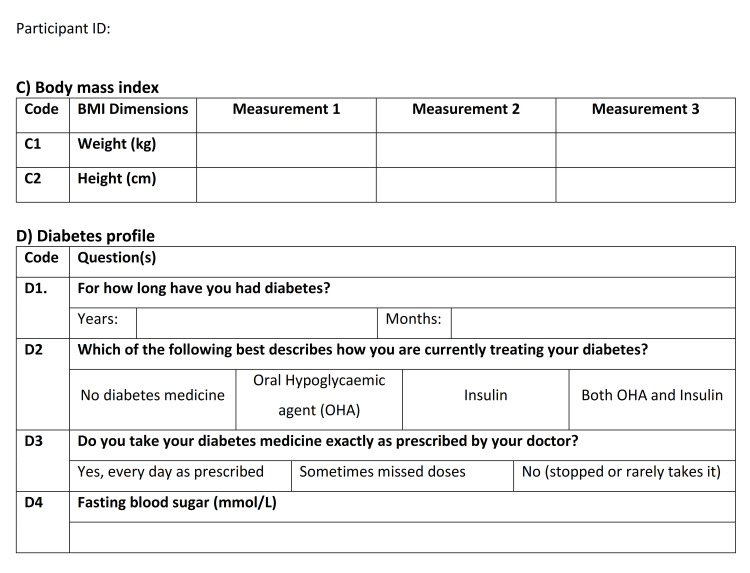
Study questionnaire page two

**Figure 6:**
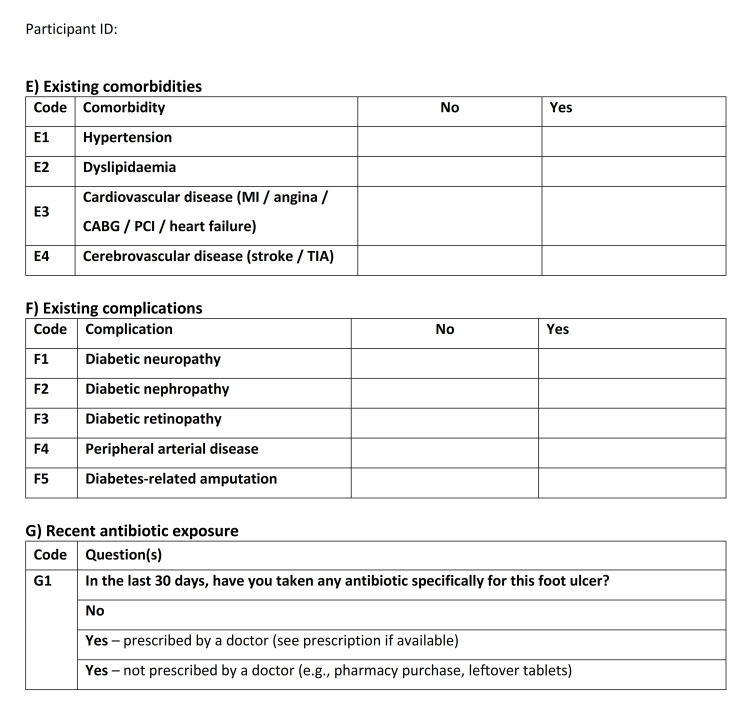
Study questionnaire page three

**Figure 7:**
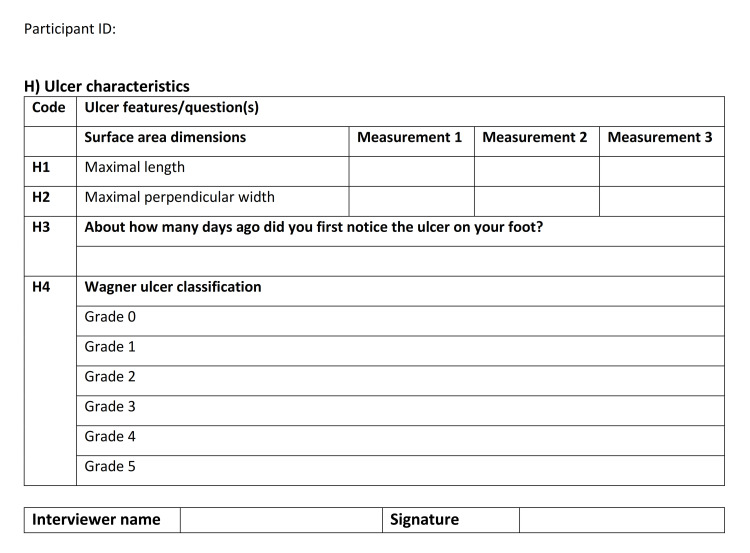
Study questionnaire page four

## Appendices

Appendix 1: study questionnaire (English version)
